# The Role of Yeast *Saccharomyces cerevisiae* in Supporting Gut Health in Horses: An Updated Review on Its Effects on Digestibility and Intestinal and Fecal Microbiota

**DOI:** 10.3390/ani12243475

**Published:** 2022-12-09

**Authors:** Vera Perricone, Silvia Sandrini, Nida Irshad, Marcello Comi, Cristina Lecchi, Giovanni Savoini, Alessandro Agazzi

**Affiliations:** 1 Department of Veterinary Medicine and Animal Sciences, University of Milan, Via dell’Università 6, 26900 Lodi, Italy; 2 Department of Human Science and Quality of Life Promotion, Università Telematica San Raffaele, Via di Val Cannuta 247, 00166 Rome, Italy

**Keywords:** horses, *S. cerevisiae*, yeast, nutrient digestibility, microbiome, gut health

## Abstract

**Simple Summary:**

In horses, a healthy state of the gastrointestinal tract (GIT) is of utmost importance to support animals’ health and performance. Several factors combine to influence the equilibrium of the GIT, including the composition of the diet, feeding management, the use of therapeutic antimicrobials, as well as biotic and abiotic stressors. Such factors can lead to an imbalance of the intestinal microbial populations and their functions and, subsequently, to reduced nutrient digestion, finally impacting the overall health of the animals. Several feed additives are used to support gut health; in horse nutrition, yeast is one of the additives most used as a supplement in the diet. Different studies showed that the addition of yeast to horse diets is able to enhance the nutrient digestibility of feeds by modulating intestinal microbial populations. Specifically, yeasts seem to act mainly upon fibrolytic and amylolytic bacteria, although, at the present moment, their role is still debated. The aim of this review is to provide the reader with an updated overview of the effects of yeast administration on nutrient digestibility and the intestinal microbial population, and to furnish an overview of yeast application in the field.

**Abstract:**

To support the overall health of horses, it is essential to maintain an optimal gut health (GH) status, which encompasses several physiological and functional aspects, including the balance and functionality of intestinal microbial populations and, accordingly, the effective digestion and absorption of nutrients. Numerous biotic and abiotic stressors can lead to an imbalance of GH, such as the quality of forages and the composition of diet, e.g., the inclusion of high energy-dense feeds to meet the energy requirements of performance horses. To support the digestive function and the intestinal microbial populations, the diet can be supplemented with feed additives, such as probiotic yeasts, that promote the ability of cellulolytic bacteria in the hindgut to digest the available fiber fractions, finally increasing feed efficiency. Among the different yeasts available, *S. cerevisiae* is the most used in horses’ nutrition; however, results of digestibility trials, as well as data on intestinal and fecal microbial populations, are sometimes contradictory. Therefore, the purpose of this review is to summarize the effects of *S. cerevisiae* on in vivo and in vitro digestibility, providing an updated overview of its effects on the intestinal and fecal microbial population.

## 1. Introduction

The horse is classified as a monogastric herbivore, with a peculiar gastrointestinal tract (GIT) organization. In the GIT of a horse, the stomach is where enzymatic digestion occurs [1], while the small intestine digests proteins, soluble carbohydrates, and fats at a pH value that is next to neutral [2]. In addition to these compartments, the large intestine (i.e., the hindgut) of the horse is the most interesting part of the GIT, since it is the fundamental site of nutrient utilization [3]. As opposed to other monogastric animals, the horse’s digestive system allows the development of huge mechanisms of dietary fiber fermentation through the degradation and utilization of cellulose and hemicellulose in the hindgut performed by specialized bacteria, leading to the final production of volatile fatty acids as an energy source [4].

The horse GIT and its microbiome play a crucial role in the animal’s health status, and their impairment can increase susceptibility to several diseases [5,6]; thus, maintaining the horse’s gut health (GH) is of primary importance for the horse and its performance. The concept of gut health includes several physiological and functional aspects, including the effectiveness of digestion and absorption of nutrients, the proper structure and function of the gut barrier, the balance and functionality of intestinal microbial populations, as well as the effectiveness of the immune system [7,8]. External factors, such as the composition of the diet and nutritional management [9], or the use of antibiotics and exposure to external stressors [10], play a role in the balance of GH.

The natural feeding behavior of horses is to continuously graze and browse, eating plant-based diets throughout the day. Therefore, fiber in forages is the main nutrient component under grazing conditions, providing energy for the maintenance requirements at least, and supporting a healthy gut environment [3]. However, the energy demands increase in horses undergoing physical exercise or during specific physiological phases (e.g., late gestating mares or lactating mares); thus, a forage-alone diet may not be sufficient to meet the energy requirements [11]. This factor is even more important when forages of poor quality, such as those with high lignin content, are used to feed the horse [12]. In the same way, non-grazing stabled horses suffer from feeding management that does not reflect their natural eating behavior and digestive physiology, being fed from two up to a maximum of four times a day, in contrast to the availability of feed all day long when on pasture. In both grazing and non-grazing horses, a portion of the diet can be replaced by increasing amounts of energy-dense feedstuffs, mainly cereal grains, depending on the workload and the energy concentration to reach. However, the inclusion of high energy-dense feeds to the detriment of forages, can result in high-starch (HS) diets (>1 g starch per kg bodyweight) [13], which are recognized to unbalance the natural environment of the hindgut, resulting in increasing concentrations of acidophilus microbes unable to digest the fiber fractions, and decreasing the hindgut pH below the optimal ranges [14,15,16]. The most common consequences of such imbalances are related to the development of microbial disturbances, metabolic disorders, and possible gut ulcerations or laminitis [17,18].

The gut microbial population can also be affected by causes other than nutritional factors and diet composition. As an example, antimicrobial agents for disease therapies in the horse are often associated with diarrhea due to a reduction in the fecal microbiome’s diversity [19,20] that causes a condition of dysbiosis; this can subsequently cause alterations in the metabolic processes of digestion and absorption of nutrients, vitamins, and amino acids biosynthesis, and regulation of the inflammatory response in the gut [19]. Moreover, the disruption of the horse’s enteric microbiota after antimicrobial treatments can cause a loss of beneficial microbes’ colonization resistance, leaving the gut more susceptible to inflammation and pathogen proliferation [21,22]. In addition to the nutritional and feeding practices and antimicrobial treatments, many external factors, including transportation, extended training, race, and social behaviors, can dramatically affect the GH of horses [23,24,25,26].

An accurate selection of feedstuffs of good quality and digestibility and the inclusion of feed additives are essential to support the digestive function and the intestinal microbial populations. Several dietary supplements that potentially exert a beneficial effect on horses’ GIT, such as probiotics and prebiotics, are commercially available. Among them, yeast supplementation has been used for years to support cellulolytic bacteria in the hindgut in the digestion of the available fiber fractions [4,13,27,28,29,30], finally increasing feed efficiency [31]. *S. cerevisiae* is the most used yeast in horses’ nutrition, with the intent to meet nutrient and energy requirements, especially in the case of poor-quality forages or HS diets [13,27]. However, the effects of *S. cerevisiae* supplementation on in vitro and in vivo digestibility, as well as its effect on intestinal and fecal microbial populations, are sometimes contradictory. Therefore, the purpose of the present review is to provide the reader with an updated overview on the role of *S. cerevisiae* in horses’ diets and the impact yeast might have on nutrient digestibility and gut microbiota.

The present review will account firstly for the effects of *S. cerevisiae* on in vitro digestibility, taking into account the type of diet fed to horses (i.e., high levels of concentrates or roughages) and focusing on gas production and fermentation kinetics. A further section will describe the effects of *S. cerevisiae* dietary supplementation on in vivo digestibility; in this section, diversities of high-forage vs. high-concentrate diets are also taken into account. In the fourth section of the manuscript, the role of *S. cerevisiae* on intestinal and fecal microbiota is evaluated, accounting mainly for the effects on fibrolytic and amylolytic bacteria, with respect to the diet fed to the animals. Finally, the last section offers some insights into the limitations of the studies currently available in the literature and provides some suggestions for future research.

## 2. Effect of *S. cerevisiae* on Nutrient Digestibility In Vitro

While the evaluation of in vivo nutrient digestibility can offer information on the effects of yeast administration, accounting for all the aspects that play a role in the GIT, this approach, however, presents some limitations, such as the biological variability among experimental subjects, the limited number of experimental replicates, the possible contamination during sample collection, and the variability in the results due to the techniques employed for the analysis [32,33]. Therefore, while some authors evaluated the efficacy of yeast administration on nutrient digestibility through in vivo experiments, some others adopted an in vitro approach. Furthermore, although, in the past, in vivo and in vitro evaluations used to be more or less independent from each other, it is of extreme importance to approach in vitro studies as a preliminary step before moving onto in vivo studies, e.g., for defining the concentration of the product to test.

The in vitro technique usually applied to evaluate the nutritive value of feed and its utilization in horses—providing information on the extent and rate at which feedstuffs are degraded—was proposed by Theodorou et al. [34]. While at first, this technique relied upon the use of rumen fluid as the source of microbial inoculum, this was later substituted by horse feces [35,36], resulting in small differences in terms of gas production (GP). Specifically, using horse feces as inoculum showed a higher lag phase than rumen fluid [35,36], which may be explained by the different number of microorganisms per gram of rumen digesta or feces [37].

Several studies evaluated the activity of *S. cerevisiae* by comparing its effects when included either in a diet with a high roughage content (HR) or a diet with a high concentrate inclusion (HC), respectively, resembling the natural feeding behavior of horses or a typical diet fed to performance horses. For the sake of this review, we refer to HR and HC diets according to the classification provided by the authors in the cited manuscripts. When not explicitly declared, we consider an HR diet as a diet with a forage:concentrate (F:C) ratio up to 70:30, while HC diets are characterized by a lower F:C ratio.

Yeast addition to the diet of horses seems to support the fermentation process, therefore improving the digestibility, as evidenced by the increased GP reported in several in vitro studies [37,38,39]. GP reflects, indeed, the physiological fermentation activity, accounting for the inoculum used and the potential of the tested additives to further stimulate the fermentative processes [39]. This effect is attributed to one of the main mechanisms of the action of yeast, which is the modulation of the gut microflora, balancing the hindgut of horses, increasing cellulolytic bacterial number, and stimulating their activity [37,38,39]. However, results reported in the literature providing evidence of the effects of yeast supplementation vary according to the type of diet fed to the animals and the concentration of the product employed.

Most of the studies evaluating the effects of yeast on in vitro fermentation reported a significant effect on GP and fermentation kinetics parameters [3,39,40], although with some differences. Recently, in a study conducted by Garber et al. [3], eight different feed substrate–fecal inoculum combinations, representative of HC and HR diets, were fermented in the presence of 11 mg/g feed substrate DM of a product containing 4% *S. cerevisiae*. The results showed an increased GP with both HC and HR feed substrates, irrespective of the substrate type and incubation ratio, while no effects were observed for fermentation rate and lag time [3]. However, it must be considered that the yeast was accounting for only 4% of the tested product; the remainder was composed of seaweed and limestone. It is, therefore, difficult to attribute the observed effect exclusively to the yeast.

Other authors found that the effect of yeast added to the inoculum varies and is strongly dependent upon multiple aspects, such as the inclusion level and the concentration of live yeast cells [39]. Elghandour et al. [39] evaluated the in vitro gas kinetics and total gas production of a diet containing 50% oat straw incubated with fecal inocula and three different commercial products of *S. cerevisiae.* Their results showed that two out of three of the tested products (2 × 10^10^ and 1.5 × 10^10^ cfu/g, respectively), at an inclusion rate of 2 mg/g DM, had higher asymptotic GP and higher GP at 12 h of fermentation compared to the control group incubated without yeast; on the other hand, the third product, which had a lower concentration of live yeast cells, showed a lower GP at different incubation hours compared to control. The authors hypothesized that such differences might be ascribed to the composition of the tested products; the three products differed in terms of the minimum guaranteed concentration of live yeast cells. This finding could suggest that for an inclusion rate of 2 mg/g DM to be effective, the product should guarantee a concentration of at least 1.5 × 10^10^ cfu/g; therefore, when the concentration is lower, the inclusion rate should be increased.

Different responses to *S. cerevisiae* addition in terms of GP and fermentation kinetics could also be related to the chemical composition of the fermented substrate. When different substrates (both forages and concentrates) were incubated with or without yeast addition, variable results were observed [37,38]. The effects of yeast administration were greater on concentrates than on forages, but improved GP and fermentation kinetics have also been observed for forages [37,38]. *S. cerevisiae* supplementation at a dose of 4 mg/g DM increased the asymptotic GP of oat grain, soybean meal, steam-rolled corn, steam-rolled barley, wheat bran, corn stover, and oat hay, while no effects were observed for corn gluten meal, alfalfa hay, and soybean hulls [38]. Similarly, the addition of different levels of yeast (i.e., 1.25, 2.5, and 5 mg/g DM) to nine forages outlined an interaction between the feeds and the yeast dose for the asymptotic GP, the rate of GP, and the lag time [37]. Interestingly, yeast addition reduced fermentation lag time, thus reducing the time before the start of GP; this effect could be due to one of the main presumed mechanisms of the action of yeast, i.e., the respiratory activity which scavenges O_2_, that has been recognized to be toxic to anaerobe bacteria, causing inhibition of adhesion of cellulolytic bacteria to cellulose [41]. In line with previous studies, Velazquez et al. [40] evaluated the fermentation kinetics of three different rations where steam-rolled corn was partially replaced either by soybean hulls or prickly pear cactus at a rate of 0, 7.5, or 15%, and incubated with *S. cerevisiae* at a dose of 0, 2, or 4 mg/g DM. The results revealed that the effect of the yeast differed between rations and was dose-dependent. Yeast quadratically decreased the asymptotic GP when corn was replaced at 15%; when corn was replaced at 7.5%, yeast quadratically increased the asymptotic GP.

Another aspect that must be considered when comparing results from different in vitro trials is the origin of the inoculum. If the inoculum comes from animals previously fed the same diet later used for fermentation, the bacteria in the inoculum are likely already adapted to the feed substrate; thus, better performance could be expected. This point was considered by a few studies and implemented in the experimental design [3,39], while other studies did not account for this variable [37,40,42].

The fermentation potential of the diet is not only represented by the total GP and fermentation kinetics, but also by the different gas types that are produced (H_2_, CO_2_, and CH_4_). Little information is available on the effects of yeast on CO_2_ production, and to the best of our knowledge, only a few studies evaluated this aspect. One study reported that yeast administration increased CO_2_ production, with higher concentrations when yeast was added at the dose of 2 mg/g DM [40]. Due to the large amounts of CO_2_ that are produced with the hydrolysis of fiber [40], the authors postulated that such a result could be indicative of improved fiber digestion. Alternatively, other studies reported no effects of yeast on CO_2_ production [30,39].

Varying results have been reported when considering the endpoint digestibility measurements, i.e., the fermentation profile, at the end of the in vitro fermentation process. Few studies evaluated the concentration of short-chain fatty acids (SCFAs) after incubation with or without yeast. In line with the role of yeast in modulating the cellulolytic bacteria, thus increasing fiber degradation [4,43,44], some studies reported increased acetate concentrations at the end of the fermentation [45]. Similarly, Elghandour et al. [39] found that, after 48 h of incubation, yeast supplementation increased SCFAs of an HR diet, although the authors did not differentiate the single SCFAs. On the other hand, Murray et al. [42] reported no variation in acetate, propionate, and butyrate concentration when increasing levels of yeast were added to high-temperature dried lucerne (0, 2, 4, 8, 16, and 40 g/g herbage).

The same variable results have also been reported for pH values at the end of the in vitro fermentation process [3,40,45]. Although it would be expected that yeast addition enhances the vessel pH due to the increase in lactate-utilizing bacteria, this result was reported only by Murray et al. [42], where the addition of yeast culture to high-dried lucerne resulted in increased pH. In other cases, yeast’s effect on pH swung according to the level of inclusion of the yeast and the composition of the fermentation substrate. For instance, when yeast was added to a diet containing 15% soybean hulls, yeast linearly decreased fermentation pH, while the opposite trend (i.e., linear increase of pH) was observed when soybean hulls’ concentration in the diet was 7.5% [40]. Finally, other authors reported no differences in pH due to yeast addition [3,45]. Garber et al. [3] hypothesized that the lack of significant effect on pH values could be due to the buffered medium used in the method of Theodorou et al. [34], which, therefore, did not entirely reflect the in vivo condition; however, the same result was also observed by Lattimer et al. [45], which employed a different fermentation system, i.e., the Daisy II incubator.

## 3. Effect of *S. cerevisiae* on Nutrient Digestibility In Vivo

Live yeast, and especially *S. cerevisiae*, is commonly used in horses’ nutrition, as it seems to have beneficial effects supporting nutrient digestibility [3,27,29]. However, in vivo studies evaluating live yeast efficacy on nutrient digestibility are still relatively limited, and their results are sometimes contradictory (Table 1).

One of the main mechanisms of action through which *S. cerevisiae* supplementation in the diet can improve nutrient digestibility is the modulation of the gut microbiota [4,43]. Although *S. cerevisiae* is recognized for its modulation of fibrolytic bacteria and, therefore, the main results are expected in HR diets, the literature seems to point in a partially different direction. Some studies demonstrate that the main beneficial effects of yeast supplementation, in terms of nutrient digestibility, are observed when horses are fed HC diets, rather than HR. Garber et al. [3] reported a stronger effect of the supplementation of 50 g/day of a product containing 4% live yeast (5 × 10^8^ cfu/g) on ponies’ apparent digestibility (AD) when they were fed HC than HR diets, with positive results on dry matter (DM), organic matter (OM), neutral detergent fiber (NDF), total detergent fiber, and hemicellulose. Similar findings were previously reported by Medina et al. [43]. In this case, the authors showed that the administration of 10 g *S. cerevisiae* in an HC diet increased the mean pH value in the cecum and the molar percentage of acetate in the colon, and decreasing the concentrations of lactic acid in both the cecum and the colon, and ammonia content in the cecum. On the contrary, no differences were observed when the same amount of yeast was administered to an HR diet. On a general note, findings from Medina et al. [43] on lactic acid concentration and pH suggest that yeast supplementation could allow horses to better tolerate HC diets, with lower risks to develop digestive disorders. In particular, the observed results can likely be ascribed to the mitigation of the negative effects of energy-dense diets on hindgut fibrolytic bacteria through the competitive exclusion of less desirable bacteria, thus increasing the efficiency of fibrous fraction utilization of HC diets [50]. Nevertheless, studies by other authors only partially corroborate the previous findings. When 10, 20, or 30 g *S. cerevisiae* per meal were supplemented to horses under HC diets [46], a quadratic effect for OM, CP, NDF, and ADF, and no effect for DM and starch digestibility, was observed. However, when compared to the control group, only CP apparent digestibility was affected by yeast supplementation, being reduced when 20 g of yeast per meal was supplemented into the diet. Similarly, Jouany et al. [27] reported no differences in the effectiveness of *S. cerevisiae* administration between HR and HC diets. They observed, indeed, a positive overall effect of the treatment on ADF apparent digestibility, irrespective of the diet. This might represent an interesting effect of *S. cerevisiae*, as ADF is the least digestible fiber component, negatively correlated with the DM digestibility of the diets. Thus, an increase in ADF digestibility could be reflected in an increased energy availability for the horse [51]. Finally, live yeast administration (2 g/day) to a mini-horse breed receiving a diet with a 60:40 concentrate:hay ratio also resulted in no improvement in the AD coefficients of DM, OM, CP, EE, NDF, ADF, and starch [47].

Focusing on HR diets only, results reported in the literature are quite contrasting. The administration of 4.6 × 10^10^ CFU/day of *S. cerevisiae* to mature horses in a 70:30 forage:concentrate ratio diet improved the apparent digestibility of DM, OM, NDF, and ADF, with a trend to improve CP [29,48]. Similar results were later confirmed by Garber et al. [3], where the administration of 50 g/day of a product containing 4% live yeast in an HR diet resulted in improved AD of DM, OM, CF, NDF, total dietary fiber, and gross energy, though ADF and CP digestibility were not affected. Nevertheless, these results differ from those of Grimm et al. [49], where the supplementation of 1 g/day *S. cerevisiae* in horses fed with an 80:20 forage:concentrate ratio diet and exposed to an abrupt change of hay during the trial failed to significantly affect nutrient digestibility. Similarly, Mackenthun and colleagues [52] conducted a trial to evaluate the effects of *S. cerevisiae* supplementation on AD of nutrients, with a special focus on fiber digestion. Their results showed that the administration of 1 or 3 g/day of yeast failed to improve nutrient digestibility; short-chain fatty acids, lactic acids, and pH in feces were not affected by the treatment either.

These contradictory results in response to yeast administration could be attributed to several aspects, including the different strains of *S. cerevisiae* employed in the studies, the horse breeds, the dosages applied, the number of viable cells, and the frequency and duration of administration, together with the composition of the diet. With respect to the composition of the diet, forage is, or should be, the main ingredient in the diet of horses. According to the NRC [11], a horse at maintenance should be fed between 1.5% to 2% body weight (BW) of hay, which could represent up to 100% of its diet. Therefore, given the relevance of the hay in the diet, its composition and quality also play a pivotal role when evaluating the effect of yeast addition. Grimm et al. [49] suggested that the lack of significant results in their trial could be related to the chemical composition of the hay, and especially to the excessive hemicellulose content; indeed, other authors reporting improved ADF digestibility [27,29,53] all employed forages with hemicellulose content lower than 18%.

In addition to the previously cited factor that can affect the efficacy of yeast supplementation to horses, the method applied to evaluate digestibility can influence outcomes obtained. Most of the studies evaluating the influence of *S. cerevisiae* on nutrient digestibility relied on the total collection of feces [3,27,46,47], while only Agazzi et al. [29] used a different method, based on the employment of acid insoluble ash (AIA) as indigestible markers. Total feces collection or AIA methods can lead to similar [54,55] or different results [54], depending on the rates of concentrate inclusion in the diet [54], the percentage of recovery of AIA in the feces [56], and the contamination with soil, if collecting the samples from the ground [57].

Moreover, the methodology applied to collect the samples could affect the outcome, potentially leading to misleading results. That is the case when fistulated horses are employed to collect intestinal content. One of the main mechanisms of action of yeast is associated with the reduction of oxygen, which favors the proliferation of anaerobic bacteria [41]. When samples are collected from fistulated horses, the presence itself of the cannula leads to oxygen entrance, thus creating conditions that stimulate the activity of the yeast, finally leading to more evident results. On the other hand, when samples are collected from non-fistulated animals, thus simulating normal conditions, the efficacy of the yeast and its effects on apparent digestibility seem to be lower, due to the already low concentration of oxygen [47]. Additionally, the duration of the collection period seems to be relevant [58]. As suggested by the guidance of the European Food Safety Authority (EFSA), an appropriate collection should last 5–7 days after an adaptation period of 21 days to the diet containing yeast, thus suggesting that a shorter period can lead to unreliable results. Ultimately, the nutritional conditions under which the experiment is carried out need to be considered. Indeed, it has been suggested that the efficacy of *S. cerevisiae* in improving fermentative activity could be better observed when the horses are subjected to nutritional challenges, e.g., starch overload or low nutritional quality forage [46].

## 4. Effects of *S. cerevisiae* on Intestinal and Fecal Microbial Population

Microbiota are essential for the development of the digestive and immune systems, stimulating and modulating the immune response while protecting against pathogenic microorganisms, and at the same time sustaining digestion [59]. Their stability is of extreme importance for maintaining a healthy status, and the imbalance of some microbial populations can lead to the occurrence of dysbiosis and, thus, to the development of disease [6,60].

The composition of the bacterial population of the equine intestinal tract varies greatly among compartments, especially at lower taxonomic levels [61,62]. In the stomach, almost all the anaerobic bacteria populations are represented by Lactobacillus and Streptococcus spp. (10^8^–10^9^ cfu/mL), with Lactobacilli being the most prevalent [63]. On the other hand, the small intestine contains between 10^6^ and 10^8^ viable bacteria/mL, most of them showing proteolytic activity [64]. The cecum represents the predominant tract of horses’ GIT in terms of total volume, and is where fermentation occurs. With approximately 10^9^ bacteria/g of ingesta [64], the cecum hosts mainly amylolytic, cellulolytic, glucolytic, hemicellulolytic, and lactate-fermenting bacteria [65]. Finally, in the colon reside 10^5^ to 10^8^/mL viable bacteria [63], including *Butyrivibrio fibrisolvens*, *Campylobacter lanienae*, *Clostridium barati*, *R. flavefaciens*, and *S. lutetiensis* [66]. Throughout the entire GIT, anaerobic fungi, archaea, and protozoa are also present [67]

One of the main mechanisms of action held responsible for the beneficial effects of *S. cerevisiae* in horses is the modulation of the hindgut microflora, ameliorating the capacity to digest fiber and limiting dysbiosis typically induced by HS or HC diets [68]. Such diets, normally fed to performance horses to meet their high energy requirements, lead to an increase in amylolytic bacteria at the expense of fiber-utilizing bacteria in the cecum and colon, resulting in a shift of the fermentation patterns, with higher production of lactate and propionate, ultimately lowering the pH and, thus, predisposing the animals to hindgut acidosis, metabolic disorders, and laminitis [69,70]. Yeast administration has been proposed, on the one hand, to stimulate the activity and the growth of cellulolytic bacteria, such as *Ruminococcus flavefaciens* and *Fibrobacter succinogenes*, thus improving fiber utilization and digestibility [71], and, on the other hand, in order to stabilize the populations of lactate-metabolizing bacteria, limiting lactate production and improving its utilization [72].

The effect of *S. cerevisiae* on fibrolytic bacteria is quite contradictory, especially when considering the diet fed to the animals (i.e., HC or HR) (Table 2). In horses receiving HC diets, the supplementation of the diet with a product providing 10 g (1 × 10^9^ cfu/g) of *S. cerevisiae* resulted in a reduced variation of the bacterial taxa relative abundance of some potential fiber-utilizing bacteria in the cecum (*Ruminoclostridium*) and the right ventral colon (Bacteroidales S24–7, *Prevotella,* and Lachnospiraceae NC2004) compared to horses fed the same diet without additive supplementation [59]. Similarly, Faubalidier et al. [9] reported an increase in cellulolytic bacteria in the feces of horses receiving 1 g of live yeast (corresponding to 4.87 × 10^8^ cfu/100 kg BW). On the contrary, other studies reported a negative effect of yeast administration on fibrolytic bacteria. The supplementation of an HC diet with 4 g/day live yeast *S. cerevisiae* (1 × 10^9^ cfu/g) resulted in a reduction of the relative abundance of *Fibrobacter succinogenes* in the feces of mature horses [13]. A similar result was also reported by Taran et al. [46], although only when the yeast was administered at an inclusion rate of 30 g/day (5 × 10^8^ cfu/g).

While some changes in the fiber-utilizing bacteria have been reported, other studies observed no significant effect of yeast supplementation on fecal concentrations of *Fibrobacter succinogenes* [47] or *Ruminococcus flavefaciens* [13,46,47], nor in the concentration of cellulolytic bacteria in the colon and cecum of horses receiving HC diets [4,43]. However, in some cases, although the relative abundance of cellulolytic populations was not modulated, their enzymatic activity was significantly affected and improved [4]. Therefore, it has been suggested that the beneficial effects observed following yeast administration are likely explained by an increase in the enzymatic activity of the bacteria involved in the digestion of cellulosic material rather than a direct effect on the bacteria biomass.

Focusing on horses receiving a diet with HR concentrations—thus, one that is closer to their natural feeding behavior—only a few studies reported a significant modulation of fibrolytic populations and activity by yeast administration, in line with what was observed in the in vivo digestibility trials. Recently, Garber et al. [73] reported that the supplementation of an HR diet with 50 g/day of a product containing 4% live yeast *S. cerevisiae* (5 × 10^8^ cfu/g) resulted in an increased relative abundance of fibrolytic bacteria in the feces. Specifically, they observed that the Lachnospiraceae family was one of the most abundant families, and they identified the genus *Roseburia*, which belongs to the same Lachnospiraceae family, as the biomarker of the animals receiving an HR diet supplemented with yeast. *Roseburia* is a butyrate-producing bacterium, and its increase could be associated with an increased energy supply. Supporting their findings in the modulation of fibrolytic bacteria by yeast administration. LEfSe analysis also revealed that the genus *Ruminococcus,* known for its role in the fibrolytic community of non-ruminant herbivores [75,76], was a significant taxon for horses receiving an HR diet supplemented with yeast. On the contrary, the majority of the studies reported in the literature failed to observe a significant modulation of the fibrolytic population in the feces [13,46] or the cecum and the colon [4,43,49] of horses receiving HR diets.

Several beneficial effects of *S. cerevisiae* have been observed on amylolytic, lactate-producing, and lactate-utilizing bacteria in horses fed HC diets [9,43,72,73], while basically no effects have been reported with HR diets [13,46]. Following the addition of yeast in the diet of horses receiving HC diets, several authors reported an increase in lactate-utilizing bacteria in the feces [9,72], in the cecum [4], and the colon [72]. At the same time, a reduction of lactate-producing bacteria (*Streptococcus*) has also been associated with the administration of 50 g/day of a product containing 4% live yeast *S. cerevisiae* (5 × 10^8^ cfu/g) to horses fed an HC diet [73]. In line with these results, Medina et al. [43] reported a reduction in the lactic acid-utilizing to lactic acid-producing bacteria ratio. However, in other studies, no changes in the streptococci population in the feces [13] nor in the cecum or colon [4] of horses were reported. Most of the studies observed no effect of yeast administration on Lactobacillus populations [9,46,47]; only the addition of 10 g (4.5 × 10^9^ cfu/g) of *S. cerevisiae* resulted in an increased concentration of Lactobacilli in the cecum (but not in the colon) of horses fed HC diets [4]. Medina et al. [43] reported that the administration of 10 g of *S. cerevisiae* in the diet of horses receiving HC diets led to an overall reduced variation in lactic acid concentration and pH of intestinal contents, suggesting that yeast supplementation may allow some horses to better tolerate HS diets without developing digestive disorders.

The aforementioned results suggest that live yeast administration could exert more beneficial effects when administered to HC diets. Its inclusion in diets with high concentrate or starch levels demonstrated a positive modulation of lactate-producing and lactate-utilizing bacteria, allowing the horses to better tolerate this type of diet without developing GIT disorders. On the contrary, the effect of live yeast addition to HR diets seems to be more limited, improving fiber digestibility by modulating fibrolytic populations only in a few cases.

Despite the evident importance of the microbial communities present in the entire intestinal tract of horses, the focus of the scientists evaluating the effect of *S. cerevisiae* administration to horses has been placed on the cecum and the colon (most of the time using the feces as their proxy) due to their predominant role in the digestion process, thus partially neglecting the other compartments. There is indeed a paucity of studies investigating the effect of *S. cerevisiae* on the microbiota of tracts other than the hindgut. Only Julliand et al. [74] reported that the supplementation of the diet with *S. cerevisiae* (10 × 10^10^ cfu) resulted in reduced concentrations of amylolytic bacteria and lactate-utilizing bacteria in the stomach of horses fed a high-starch diet.

An important aspect that needs to be taken into consideration when evaluating the role of *S. cerevisiae* on GIT microbial populations is the age of the animals. To demonstrate that a dietary intervention has a beneficial and lasting effect on microbiota and, therefore, on gut health, it is essential to identify the timepoint when the microbiota stabilize. GIT colonization starts in the fetus, during the intrauterine life [77], and is a dynamic process influenced mainly by the environment and management of the animals [78]. At birth, the foal acquires several microorganisms that form its initial microbiota [79]. Such microorganisms are mainly derived from those present in the feces, vagina, and the material environment, and are mainly represented by the phyla Firmicutes and Proteobacteria, along with the phylum Bacteroidetes [80,81]. The foal’s microbiota gradually changes over the first days of life, with an increasing abundance of fiber fermenting bacteria, starting approximately 7 days after birth [81,82]. This change is driven by some typical behaviors, such as coprophagy, along with the start of the consumption of forage and concentrate [77,83]. At this point, Firmicutes is the most abundant genera [79,80]. At day 20 of life, the microbiota seem to be more uniform, although their composition is still significantly different from that of foals aged 50 days and older [82]. The microbiota continue adapting and changing until they reach a certain stability at around 50–60 days of life [80,82]. Although more stable, the microbiota continue to differ from that of mature horses for as long as 9 months [68].

This indicates that the period between birth and 50–60 days is the most suitable window of opportunity for permanently influencing the microbiota. Unfortunately, studies evaluating the employment of yeast at such a young age are very scarce, and mainly related to the administration of yeast to the mares during the gestation phase, rather than to the foals [84,85]. The studies evidenced that the addition of live yeast to the mare’s diet was able to influence the microbial profile of the foal’s hindgut, limiting the variations in the microbial profile and, thus, potentially buffering the shifts in the microbial ecosystem [84,85]. On the other hand, studies conducted on the employment of probiotics other than yeast in foals led to negative results, where the foals receiving the probiotic were sometimes more likely to present diarrhea, along with depression, colic, and anorexia, compared to the animals receiving a control diet [86].

Most studies investigating the impact of yeast administration on the intestinal microbial population of horses evaluated only specific bacteria or groups of bacteria (mainly cellulolytic, lactate-producing, and lactate-utilizing bacteria) using traditional culturing techniques [4,43,72]. Only more recently, a deeper approach using next generation sequencing (NGS) techniques was performed, producing a broader illustration of the effects of yeast administration on the intestinal microbiome. These studies confirmed *Firmicutes* and *Bacteroidetes* as the two dominant phyla in the feces [72,73,87] as well as in the cecum and colon [72] of horses, following the available literature [88,89]. This result was corroborated by microbial diversity analysis, which revealed no differences (Chao1, observed OUT, and Shannon indexes) in the feces [73,87], nor in the cecum or colon, when yeast was administered [72]. Only Grimm et al. [72] reported that the fecal diversity of horses receiving a product providing 10 g (1 × 10^9^ cfu/g) of yeast *S. cerevisiae* increased compared to control animals, irrespective of the composition of the diet (HC or HR). Similarly, Faubaldier et al. [9] reported a slight increase in fecal bacteria diversity, as shown by the greater number of molecular species identified through temporal temperature gradient gel electrophoresis (TTGE). Interestingly, Lucassen et al. [87] observed significant differences in the alpha diversity of fecal microbiota between horses receiving a control diet and those receiving a diet supplemented with a *S. cerevisiae* fermentation product only after the animals were vaccinated against influenza. The authors speculated that yeast administration altered the vaccination-induced spectrum of released cytokines and other mediators, potentially affecting the gut microbiome [87].

Studies based on 16S rRNA analysis of the intestinal and fecal microbiome of horses revealed great individual variability. Lucassen et al. [87] observed that the bacterial communities were separated more clearly by individual animals rather than by yeast administration, thus suggesting that the individual animal has the highest impact on the fecal microbiota composition. The same results were also confirmed when analyzing the differentially abundant taxa between supplemented and non-supplemented animals; the significant OTUs seemed to be more influenced by the animals themselves rather than by the yeast [87]. Similar results were also reported by other studies [13,73], which suggest that for an optimal evaluation of the effects of yeasts or any other products on horses’ microbiome, each animal should serve as its own control to avoid biases due to biological variability.

Aside from the individual variability, the highly variable results reported in the literature, in terms of the effect of yeast supplementation on microbial population, could have several possible explanations, including the type of sample on which the analysis was carried out. Most of the time, the analysis of the microbial populations is carried out on fecal samples, representing a non-invasive and less labor-intensive approach compared to the collection of the cecum or colon content using fistulated horses. However, it has been suggested that the feces might not be a good proxy of what is happening in the hindgut, given the distance between the cecum, the right ventral colon, and the rectal ampulla [72]. Another possible reason for the differences in the observed results could be the yeast strain and its ability to resist the gastrointestinal tract. If the yeast reaches the hindgut in a viable concentration, it can promote a probiotic effect. On the other hand, if the yeast, inactivated in the stomach due to the acid pH, is not able to reach the hindgut in a viable form, instead of exerting a probiotic activity it could exert a prebiotic activity, given its composition and the high concentration of glucans and mannans, which serve as fermentation substrate for the intestinal microbiome [46]. Last but not least, the ability of the yeast to colonize the hindgut should also be analyzed. Different authors reported the presence of live yeast cells in the intestinal content as well as in the feces of horses receiving dietary supplementation of live yeast, thus suggesting their ability to survive throughout the digestive tract of horses [4,9,43]. However, after the end of the supplementation, no more yeast was found in the aforementioned compartments, suggesting the yeast cannot colonize and establish in the large intestine [4,9].

## 5. Future Perspectives and Conclusions

The main mechanism of action attributed to *S. cerevisiae* as a probiotic feed additive is the modulation of the gastrointestinal microflora, associated with its scavenging activity towards O_2_ [41]. *S. cerevisiae* is believed to support the activity and the growth of cellulolytic bacteria, thus improving fiber utilization and digestibility [53], while stabilizing the populations of lactate-metabolizing bacteria, limiting lactate production and improving its utilization [72].

However, it appears clear from this literature review that, while the addition of *S. cerevisiae* to the diet of horses could somehow benefit gut health, e.g., improving, in some cases, the nutrient digestibility or modulating fibrolytic and amylolytic bacteria, its efficacy and results are quite contradictory and highly variable. Such variability could be attributed to several factors, such as the strain, the dose, and the concentration of the yeast employed, the duration of the administration, the composition of the diet, as well as implicit features of the horse, such as age. Unfortunately, due to the marked differences among the available studies in the literature, it is difficult to identify which of these aspects could have a predominant role. Therefore, we encourage a meta-analysis to shed light on this very important aspect.

Changes in the activity of the horse microbiota may affect the health of the digestive system and may lead to the development of several diseases. Generally, more research is still required to fully comprehend the impact of *S. cerevisiae* on the gut microbiota. To highlight the significant changes that can occur in a horse’s GIT, a nutrigenomic approach, paying close attention to the microbiome, metabolome, or proteome, should be the first choice. Omics techniques applied in-depth to horses could improve the knowledge in this field, elucidating the molecular mechanisms and modifications occurring in horses’ GITs and identifying their influence on the entire animal. At the same time, omics techniques could help to assess the effect of *S. cerevisiae* dietary supplementation, not merely on resident microbial population balance and changes, but also accounting for generated metabolites and active molecules that can affect, either positively or negatively, the function of the GIT and local and systemic immune responses.

One of the main limits that emerges from this review is that almost the totality of the studies evaluating the role of *S. cerevisiae* on gut microbiota of horses focused on the hindgut, most of the time using the feces as a proxy of the distal colon. The degree to which feces can be representative of the hindgut environment is, however, still debated. Furthermore, there is a significant lack of information on the effect of *S. cerevisiae* administration on the microbiota composition of GIT compartments other than the hindgut. The ability of *S. cerevisiae* to overcome the stomach must be evaluated. If this can occur, the entity in which it occurs must be determined. Modulations of the microbiota in response to yeast administration should also be considered in the proximal intestine. For instance, the duodenum, jejunum, and ileum are the main target sites of different pathogens, such as rotaviruses. Therefore, knowing the effect of *S. cerevisiae* in these locations could allow for a more appropriate employment of yeast to restore the normal balance of the intestine. This also leads us to highlight the lack of studies investigating the administration of *S. cerevisiae* in horses experiencing gastrointestinal diseases, and the lack of models that serve this purpose.

One aspect that has also been neglected is the evaluation of the kinetic changes of intestinal microbiota in the course of *S. cerevisiae* administration. There is indeed no available data on how long is required for the yeast to modify the gut microbiota composition, and how long the modifications will last. Furthermore, having recognized the time window between birth and 60 days of life as the most suitable window of opportunity for inducing long-lasting effects on the microbiota, we encourage studies on the administration of *S. cerevisiae* in foals.

Besides the effects of live yeast *S. cerevisiae* on microbial population and digestibility of nutrients, some more recent studies showed the potential benefits of other yeast-derived products, i.e., *S. cerevisiae* fermentation products (SCFPs). SCFPs are unique micro-ecological products that are composed of yeast biomass (including some residual viable cells, dead cells, and yeast cell wall fragments), fermentation metabolites, and residual growth medium. Research showed how SCFP, as a nutraceutical fed to horses, produced an immunomodulatory effect that was independent of the gastrointestinal tract’s major microbiota modifications [87]. In monogastric animals and ruminants, as well as chickens, SCFPs were able to produce a direct interaction of pre- and postbiotic ingredients with local gut epithelial [90,91] and immune cells [92], modulating both systemic and mucosal immune responses [93]. Preliminary information on dietary SCFPs in horses evidenced a stress-mitigating effect when young horses were subjected to prolonged exercise [94,95]; this is an additional main point of interest to be further elucidated, with respect to how SCPF-mediated pathways can affect and regulate inflammatory responses in horses.

## Figures and Tables

**Table 1:** Effects of *S. cerevisiae* on in vivo apparent digestibility (AD).

Diet Composition	Inclusion Rate	Yeast Concentration (cfu/g)	Effects	Reference
HC	50 g/day of a product containing 4% yeast	5 × 10^8^	↑ DM, OM, NDF, total detergent fiber, and hemicellulose AD	[3]
HC	10 g/day	4.5 × 10^9^	↑ cecal pH and colon acetate ↓ cecal and colon lactic acid; ↓ ammonia concentration	[43]
HC	10, 20, or 30 g/meal	5 × 10^8^	↓ CP AD with 20 g supplementation	[46]
HC	2 g/day	20 × 10^9^	No changes on DM, OM, CP, EE, NDF, ADF, and starch AD	[47]
HC and HR	10 g/day	4.5 × 10^9^	↑ ADF AD	[27]
HR	50 g/day	4.6 × 10^10^	↑ DM, OM, NDF, ADF AD	[29,48]
HR	50 g/day of a product containing 4% yeast	5 × 10^8^	↑ DM, OM, CF, NDF, total dietary fiber, and gross energy AD	[3]
HR	1 g/day	1 × 10^9^	No effects on DM, OM, CP, NDF, ADF, and lignin AD	[49]

HR, high-roughage diet; HC, high-concentrate diet; DM, dry matter; OM, organic matter; NDF, neutral detergent fiber; ADF, acid detergent fiber; ADL, acid detergent lignin; CP, crude protein; EE, ether extract; CF, crude fiber; AD, apparent digestibility; ↑, increased; ↓, decreased.

**Table 2:** Effects of *S. cerevisiae* on intestinal and fecal microbial population of horses.

Diet Composition	Inclusion Rate	Yeast Concentration (cfu/g)	Effects	Type of Sample	Reference
**Effects on fibrolytic bacteria**					
HC	10 g/day	1 × 10^9^	↓ variation *Ruminoclostridium*	Cecum content	[72]
HC	10 g/day	1 × 10^9^	↓ variation Bacteroidales S24–7, *Prevotella,* and Lachnospiraceae NC2004	Colon content	[72]
HC	1 g/day	4.87 × 10^9^ *	↑ in cellulolytic bacteria	Feces	[9]
HC	4 g/day	1 × 10^9^	↓ *Fibrobacter succinogenes*No effects on *Ruminococcus flavefaciens*	Feces	[13]
HC	2 g/day	20 × 10^9^	No effects on *Fibrobacter succinogenes* and *Ruminococcus flavefaciens*	Feces	[47]
HC	10 g/day	4.5 × 10^9^	No effect on cellulolytic bacteria	Cecum and colon content	[4,43]
HR and HC	10, 20, and 30 g/day	5 × 10^8^	↓ *Fibrobacter succinogenes* only with 30 g/day. No effects at lower inclusion rate No effects on *Ruminococcus flavefaciens*	Feces	[46]
HR	50 g/day of a product containing 4% yeast	5 × 10^8^	↑ relative abundance of fibrolytic bacteria	Feces	[73]
HR	1 to 30 g/day	5 × 10^8^ to 4.5 × 10^9^	No results	Feces, cecum and colon content	[4,13,43,46,49]
**Effects on amylolytic, lactate-producing, and lactate-utilizing bacteria**					
HC	1 to 10 g/day	1 × 10^9^ to 4.87 × 10^9^	↑ lactate-utilizing bacteria	Feces, cecum and colon content	[4,9,72]
HC	2 g/day of a product containing 4% live yeast	5 × 10^8^	↓ of lactate-producing bacteria (*Streptococcus*)	Feces	[73]
HC	10 g/day	4.5 × 10^9^	↓ lactic acid-utilizing:lactic-producing bacteria	Cecum and colon content	[43]
HC	1 g/day	10 × 10^10^	↓ amylolytic bacteria and lactate-utilizing bacteria	Stomach content	[74]
HC	10 g/day	4.5 × 10^9^	↑ *Lactobacilli*	Cecum content	[4]
HC and HR	1 to 30 g/day	5 × 10^8^ to 20 × 10^9^	No effects on lactobacillus population	Feces, cecum, and colon content	[9,46,47]

HR, high-roughage diet; HC, high-concentrate diet; * cfu/100 kg BW; ↑, increased; ↓, decreased.

## Data Availability

Not applicable.
